# Research Progress on Nutritional Components, Functional Active Components, and Pharmacological Properties of *Floccularia luteovirens*

**DOI:** 10.3390/cimb47090742

**Published:** 2025-09-10

**Authors:** Siyuan Gou, Lihua Tang, Huange Huang, Yanqing Ni, Tongjia Shi, Wensheng Li, Yan Wan, Xu Zhao

**Affiliations:** 1 Institute of Urban Agriculture, Chinese Academy of Agricultural Sciences, Chengdu 610299, China; 2 College of Food and Biological Engineering, Chengdu University, Chengdu 610106, China; 3 Chengdu National Agricultural Science and Technology Center, Chengdu 610299, China; 4 The Edible Fungi Research Institute of Shanghai Academy of Agricultural Sciences, Shanghai 201403, China; 5 Key Laboratory of Coarse Cereal Processing, Ministry of Agriculture and Rural Affairs, College of Food and Biological Engineering, Chengdu University, Chengdu 610106, China

**Keywords:** edible and medicinal fungi, *Floccularia luteovirens*, nutritional components, functional active substances, pharmacological properties

## Abstract

Edible and medicinal fungi are a general term for large fungi with both edible and medicinal values. As a unique wild edible and medicinal fungus in the Qinghai-Tibet Plateau, the ‘Four Medical Classics’ of the Tang Dynasty has recorded *Floccularia luteovirens* effects of external application and internal administration on swelling, cold disease, and neck stiffness. At present, it has not been artificially domesticated and has significant development potential. The mushroom is rich in nutrients. The crude protein content of 100 g dried product is 33~39% (up to 38.71 g, about 2.2 times that of *Flammulina velutipes*). It contains 19 amino acids (including 8 essential amino acids for the human body; tryptophan accounts for 21.55~22.63%). It is also rich in minerals such as selenium, zinc (0.09 g/kg), and iron (0.3 g/kg) and vitamins B1 (0.10 mg), B2 (1.10 mg), C (4.50 mg), and E (6.20 mg). Among the functional active substances, polysaccharides (containing 20.1% β-glucan and 5.7% mannan-oligosaccharide) had antioxidant and immunomodulatory effects, which could alleviate the weight loss of diabetic rats. The IC50 of DPPH free radical scavenging rate of phenolics (ferulic acid, etc.; total phenolic content of 4.21 ± 0.06 mg/g) was 43.85 μg/mL; there was also adenosine, volatile oil, and other components. Pharmacologically, the DPPH free radical scavenging rate of the extract was 65 ± 0.46%, the tumor inhibition rate of the polysaccharide on the tumor-bearing mice was 42.48%, the gastrodin was biocatalyzed (conversion rate 85.2%), and the extracellular polysaccharide could inhibit the color change in shrimp to achieve preservation. This paper reviews its related research progress and provides a reference for its development in the fields of healthy food and biomedicine.

## 1. Introduction

**Figure 1:**
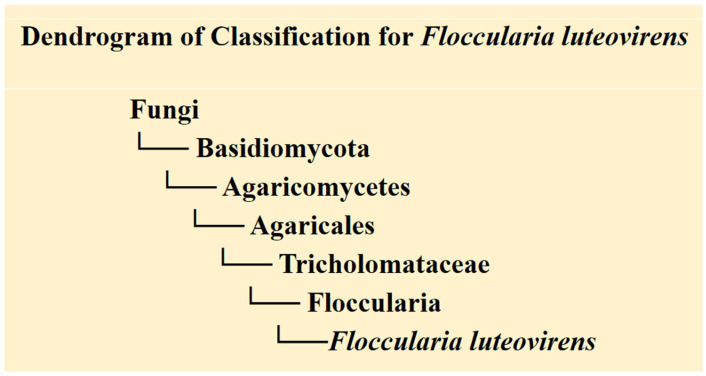
Dendrogram of classification for *Floccularia luteovirens*.

*Floccularia luteovirens* belongs to Basidiomycota, Agaricomycetes, Agaricales, Tricholomataceae, and Floccularia [1] (Figure 1). It is a rare edible and medicinal fungus unique to the Qinghai-Tibet Plateau [2]. It is known as the ‘Treasure of the Prairie’ [3]. It mainly lives on the Kobresia meadow at an altitude of 3200–4800 m [4]. Up to now, relevant geographical distribution reports have shown that it is distributed in Qinghai, Tibet, Sichuan, Gansu, and other provinces [5]. A comprehensive and systematic investigation by Xie Zhanling et al. showed that the distribution latitude range of the mushroom in China was N 28°29′~37°69′, and the longitude range was E 90°4′~102°1′ [5]. The nutritional components of *Floccularia luteovirens* are very rich, including sugars, proteins, fats, vitamins, and minerals [6], and it is rich in amino acids [7].

The fruiting body of *Floccularia luteovirens* (*F. luteovirens*) is thick and tender, rich in aroma, rich in nutrition, and has medicinal effects [6]. As a traditional Tibetan medicine, its medicinal value has been recorded since the Tang Dynasty [8,9,10,11]. According to the records in the ‘Four Medical Classics’, *F. luteovirens* and eggs, purple grass, and other agents, external application and oral administration, can eliminate swelling, and egg yolk, cattle urine, and other preparations of hot compress can be used to treat colds; at the same time, eating and using mushroom soup to iron the back of the neck can treat neck stiffness. The records also indicate that eating *F. luteovirens* fried in mustard oil and taking cold stone water before eating *F. luteovirens* will cause poisoning [8]. According to the herdsmen, it mainly has the effect of preventing and treating colds [10]. Although research on *F. luteovirens* started late in China, many studies have found that it is not only rich in a variety of nutrients but also has clinical efficacy for many diseases [12], attracting more and more scholars’ attention, thus becoming a research hot spot. At present, most of the review studies on *F. luteovirens* focus on ‘panoramic combing’, focusing on the integration of discrete information scattered in the fields of taxonomy, ecology, cultivation techniques, and so on, forming a comprehensive overview of its research history and current situation, which is essentially a ‘broad summary’ of existing research results. In this paper, the research perspective and content focus have achieved significant innovation: the research focus is highly focused on the core dimension of the ‘edible and medicinal value’ of *F. luteovirens*, abandoning the balanced presentation of multi-domain information and focusing on its value attributes. In this paper, related research on the three progressive levels of ‘nutrient composition (basic value support), active substance (core functional carrier), and pharmacological characteristics (practical application potential)’ was reviewed, which filled the research gap of the value dimension of the mushroom being ‘tasteless and lack of system’ in the previous review. It provides a more directional reference for subsequent industrialization research, which is also a key direction that has not been covered by previous comprehensive reviews.

## 2. Nutrient Components of *F. luteovirens*

### 2.1. Proteins and Amino Acids

The crude protein content of 100 g dried *F. luteovirens* can be as high as 38.71 g [9], and the average protein content is between 33% and 39%, which is about 2.2 times the crude protein content of *Flammulina velutipes* [13]. There are differences in protein content between different geographical and regional samples [7,14,15,16], which may be affected by endogenous factors (such as genetic differences) and exogenous factors (such as light, altitude, temperature, and soil conditions). The amino acids of *F. luteovirens* were rich in 19 kinds of amino acids (Table 1), including 8 kinds of essential amino acids and 11 kinds of non-essential amino acids. The content of tryptophan accounted for 21.55–22.63% of the total amino acid content. Asparagine, which was not detected in Qinghai *F. luteovirens*, was detected in Naqu *F. luteovirens* in Tibet, and the content accounted for 9.72~12.77% of the total amino acid content [7]. There is one more amino acid in *F. luteovirens* than the Shiitake amino acid species on the table, but the Shiitake contains only 7 of the 8 essential amino acids in the human body [17]; at the same time, 18 amino acids are also more than the type commonly reported in soybean protein analysis [18]. Therefore, it is speculated that *F. luteovirens* will also be a good ‘plant-based meat alternative’ [19].

### 2.2. Minerals

*F. luteovirens* is rich in essential minerals for the human body (Table 2), especially with respect to the lack of zinc, copper, manganese, and other high contents in most foods. The content of calcium is up to 0.5 g/kg, and the content of zinc is about 0.09 g/kg, which is less than in other foods. The content of iron is also high, up to 0.3 g/kg [15]; phosphorus is 10.39~11.89 g/kg; potassium is 28.61~32.43 g/kg [20,21], which is 10~15 times higher than that of plant food. In particular, the content of ‘selenium’, the nemesis of cancer, is very high, with 0.0382 mg in 100 g of dry product [3,22]. Calcium is an important component of human bones and teeth, and it can regulate the excitability of the heart, nerves, and muscles. Iron is involved in the composition of hemoglobin in the human body and plays an important role in the normal transport of oxygen and carbon dioxide. Zinc is a cofactor of many enzymes. It participates in the synthesis of nucleic acid proteins through enzymes, promotes human growth and development, and enhances immunity. Copper is an activator of human hemoglobin. Phosphorus is involved in the composition of enzymes, which can activate substances, and is also an important component of bone composition. Selenium participates in the catalytic reaction of various enzymes, especially the antioxidant effect of glutathione peroxidase in red blood cells. It also promotes the production of immunoglobulin and protects the integrity of phagocytes. It is a trace element with good anti-cancer and anti-inflammatory effects [22].

### 2.3. Vitamins

The vitamins in *F. luteovirens* are vitamin B_1_ (0.10 mg), vitamin B_2_ (1.10 mg), vitamin C (4.50 mg), and vitamin E (6.20 mg). It is also rich in carotenoids that can be converted into vitamin A (1.61 mg) in the human body (Table 3) [23]. When compared to the Recommended Daily Intake (RDI) for adults (based on references for dietary nutrient intake, vitamin B_1_ accounts for approximately 8.3% of the RDI (1.2 mg/day for adult males). Vitamin B_1_ can improve the normal digestive function of people and has obvious effects on the prevention and treatment of dyspepsia and loss of appetite. Vitamin B_2_ makes up around 84.6% of the RDI (1.3 mg/day for adult males). This vitamin can make the skin cells in the human body grow and develop well and prevent and improve the occurrence and development of skin diseases, such as skin inflammation, angular cheilitis, cheilitis, and glossitis. Vitamin C constitutes about 5.0% of the RDI (90 mg/day for adults). Vitamin C can effectively prevent the occurrence of various chronic diseases, such as cardiovascular disease, cancer, etc., and can delay the speed of human aging [9]; vitamin E, as α-tocopherol equivalent, represents roughly 41.3% of the RDI (15 mg/day for adults). It has physiological activities, such as enhancing immunity and disease resistance. Carotenoids (1.61 mg) are converted into vitamin A at a typical conversion factor of 12 μg carotenoids ≈ 1 μg retinol activity equivalents (RAEs) [24]. This yields approximately 134.2 μg RAE of vitamin A, accounting for 14.9% of the RDI (900 μg RAE/day for adult males). Carotene has the effect of preventing night blindness and maintaining mucosal health.

### 2.4. Other Nutrients

At present, there are few studies on crude fiber, crude fat, and nitrogen-free extract in *F. luteovirens*. At present, only a single datum shows that, in 100 g dry product of *F. luteovirens*, crude fiber is 8.04 g [3], nitrogen-free leachable is 25.13 g, and crude fat is as high as 8.66~15.28 g [9,14].

In summary (Table 3), as a high-protein, multi-mineral, and vitamin-rich characteristic fungus, *F. luteovirens* has both nutritional and functional characteristics, and has the potential to become a new ‘plant meat’ resource. In the future, we will explore its multiple values in healthy food and functional agriculture and ecological economy and provide new choices for the field of food nutrition and health.

**Table 3:** List of nutritional components and functional characteristics of *F. luteovirens*.

Nutritive Classification	Specific Composition and Content (100 g Dry Product)	Functions and Features	Reference
protein and amino acids	Crude protein: 33~39% (dry product about 38.71 g), 2.2 times that of *F. luteovirens*	The types of amino acids were better than those of mushrooms (less than 1 essential amino acid) and soybeans (less than 1 amino acid). It was speculated that it could be used as ‘plant meat’.	[13]
	Amino acids: 19 kinds (including 8 kinds of essential amino acids and 11 kinds of non-essential amino acids).		[7,17]
	Tryptophan accounted for 21.55~22.63%, and Tibetan samples contained asparagine (9.72~12.77%).		
mineral matter	Calcium: 0.5 g/kg	Constitutes bone teeth, regulates neuromuscular excitability	[15]
	zinc: 0.09 g/kg	Enzyme cofactors promote growth and development and enhance immunity	
	iron: 0.3 g/kg	Involved in hemoglobin synthesis and transport of oxygen and carbon dioxide	
	phosphorus: 10.39~11.89 g/kg	Participates in enzyme composition and constitutes the bones	[20,21]
	potassium: 28.61~32.43 g/kg	By promoting the discharge of sodium, it helps to regulate blood pressure and is beneficial to cardiovascular health.	
	Selenium: high content 0.0382 mg in 100 g of dry product	Antioxidant, anti-cancer, promotes immunoglobulin production	[3,22]
vitamin	Vitamin B_1_: 0.10 mg	Promotes digestion, improves loss of appetite	[23]
	Vitamin B_2_: 1.10 mg	Maintain skin and mucosal health, prevents inflammation	
	Vitamin C: 4.50 mg	Antioxidation, prevention of cardiovascular disease, cancer, and anti-aging	[23]
	Vitamin E: 6.20 mg	Enhance immunity and disease resistance	
	Carotene: 1.61 mg (can be converted to vitamin A)	Prevents night blindness, protects the mucosa	
other components	crude fiber: 8.04 g	At present, there are few research data, which need to be further excavated.	[3]
	crude fat: 8.66~15.28 g		[9,14]
	nitrogen-free extract: 25.13 g		

## 3. Functional Active Substances of *F. luteovirens*

### 3.1. Polysaccharides: The First Item

Studies have shown that polysaccharides are one of the important components in *F. luteovirens*, and they have pharmacological activities such as antioxidant, anti-inflammatory, anti-tumor, anti-aging, analgesic, and immune regulation. The reported polysaccharides include glucose [25], reducing sugar [15], mannose oligosaccharides [26], mannose [25], fucose [25], rhamnose [25], galactose [25], xylose [27,28], arabinose [27,28], and β-glucan [26]. The extraction methods of polysaccharides from *F. luteovirens* are ultrasonic extraction, water extraction, organic solvent extraction, and column separation.

The optimum conditions for ultrasonic-assisted extraction of polysaccharides from *F. luteovirens* fruiting bodies were optimized by Du et al. [29], and the extraction rate of polysaccharides was 7.93%. Shi Qiangqiang [30] used a DEAE-52 cellulose chromatography column to separate and purify the polysaccharide from the mycelium of *F. luteovirens*, and the polysaccharide content reached more than 99%. Liu Yang [26] used high-performance liquid chromatography (HPLC) to detect the content of β-glucan and mannan oligosaccharides in the polysaccharide of *F. luteovirens*. The content of β-glucan and mannan oligosaccharides was 20.1% and 5.7%, respectively. The content of polysaccharides in the water extract of *F. luteovirens* mycelia was determined by a phenol-sulfuric acid method [31], which was 31.21%. Tao Yanduo [32] determined the production process route of the polysaccharide extract of Floccularia L, which showed that the polysaccharide content was more than 53%, and the mycelium of *F. luteovirens* was successfully cultured. The polysaccharide content was 31%, and the yield was 2.3%. Wang Hong [33] determined that the optimal refining medium for the extracellular polysaccharide of *F. luteovirens* was potato 20 g, glucose 4 g, yeast extract 0.20 g, KH_2_PO_4_ 0.15 g, MgSO_4_ 0.05 g per 100 mL, VB_1_12 μg per 1 mL, and pH 6.0. Xiao Qianqing [34] systematically established the detection method of *F. luteovirens* polysaccharides and optimized the extraction and fermentation process. The results showed that the extraction rate of polysaccharides was 11.86% under the optimal conditions. Liu et al. [27] extracted with hot water at 80 °C, deproteinized by the Sevag method, decolored by H_2_O_2_, and precipitated by 95% ethanol to obtain crude polysaccharide, and the yield was 1.72%. Wang Huan et al. [35] analyzed the system composition of the crude polysaccharide extracted and separated from *F. luteovirens* and obtained a total sugar content of 65.0% and a reducing sugar content of 7.2%.

Based on the data in Table 4, the efficiency of *F. luteovirens* polysaccharide-related methods can be evaluated from two core perspectives: extraction yield (for raw material processing) and purity (for downstream application).

**Figure 2:**
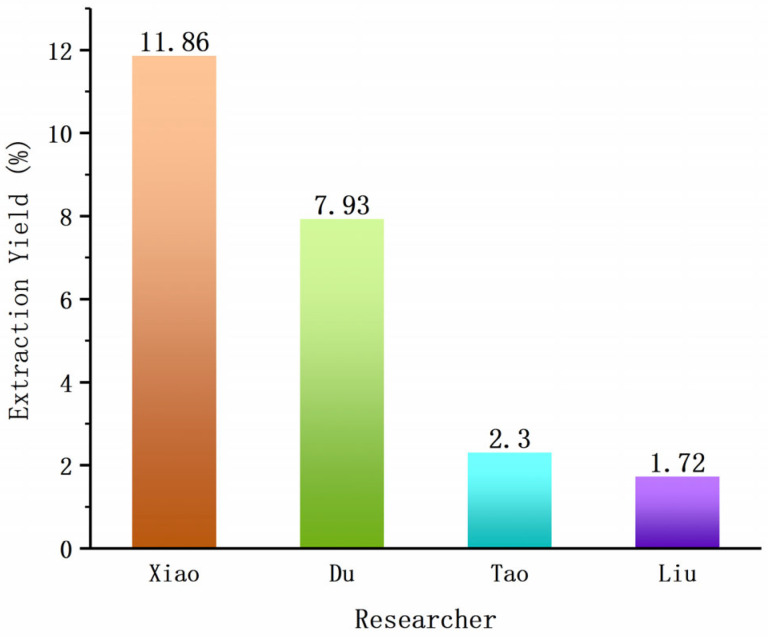
Extraction rate difference diagram.

With respect to extraction yield, Xiao’s optimized process is the most efficient (Figure 2). Shi’s DEAE-52 purification enables ultra-high purity. In terms of special cases, there is fermentation for extracellular polysaccharides. Wang Hong’s [33] medium optimization for extracellular polysaccharides (EPSs) provides a complementary approach to raw material extraction. Although no direct EPS yield was reported, the optimized medium (e.g., glucose as a carbon source and yeast extract as a nitrogen source) creates conditions for efficient EPS synthesis by *F. luteovirens* mycelia, which could be a promising alternative to extracting polysaccharides from natural fruiting bodies (especially when fruiting bodies are scarce).

### 3.2. Volatile Organic Compounds (VOCs)

The volatile oil category in *F. luteovirens* is also very rich. Su et al. [36] identified alkanes, esters, ketones, acids, aldehydes, etc. from the volatile oil of *F. luteovirens*. There are 11 main components, including butyl-diethylborane (4.26%), 2-methyldodecane (4.87%), cyclopentane undecanoate methyl ester (3.24%), palmitic acid (7.87%), n-hexadecane (6.74%), hexylcyclopentane (5.43%), n-heptadecane (6.24%), 2.9-dimethyldecane (8.42%), linoleic acid methyl ester (10.94%), 2-methyl eicosane (12.23%), and 7-hexyl eicosane (10.57%). Wei Yongsheng [37] et al. studied the volatile components of the fruiting bodies of *F. luteovirens* by headspace solid-phase microextraction–gas chromatography–mass spectrometry (SPME-GC-MS). The main volatile components were 3,7-dimethyldecane (13.89%), zingiberene (9.10%), 2,3-dimethyldodecane (8.76%), bergapten (4.62%), octadecane (3.65%), 2-nonanone (3.41%), 2,3,5,8-Tetramethyldecane (2.89%), curcumene (2.83%), 6-octen-2-one (2.80%), and β-bisabolene (2.42%). It also shows that the fruiting body of *F. luteovirens* has a strong fragrance, and its main volatile chemical composition is various terpene compounds with molecular formulas C15H24 and C15H22, various alkane isomers with molecular formulas C14H30, C12H26, and C18H38, as well as various ketene and aldehyde alcohols containing C8, C9, and C13. Zhou Jinsong et al. [38,39] confirmed 13 kinds of volatile oil components in the fruiting body of *F. luteovirens*, and the identified components accounted for 97.1% of the total volatile oil content. The main component was unsaturated fatty acids.

These volatile oil components are not only of great value for flavor enhancement: terpenoids (such as zingiberene, β-bisabolene), specific alkanes (such as 3,7-dimethyldecane), and esters (such as methyl cyclopentane undecanoate) together shape the strong and unique aroma profile of *F. luteovirens*, providing potential for its application in the food industry (e.g., development of natural condiments and functional foods)—relying on the properties of natural flavor substances, they can enhance the sensory appeal and market competitiveness of food.

At the same time, many components show potential value in the field of drug development: for example, bergapten (a furanocoumarin) has been proven to have photobiological activity and pharmacological potentials, such as antibacterial and anti-cancer effects; unsaturated fatty acid methyl esters (such as methyl linoleate) participate in lipid metabolism in the body and have biological effects of anti-inflammation and blood lipid regulation; terpenoids and alkane derivatives also often exhibit antioxidant, antibacterial, and other activities, providing a theoretical basis for *F. luteovirens* as a medicinal fungal resource and for the screening of lead compounds for natural drugs.

Note: the fatty acid derivatives identified in this section are volatile esters (e.g., methyl linoleate) detected under SPME conditions, which differ from the total fatty acids analyzed in Section 3.4.

### 3.3. Other Compounds in Fruiting Bodies

Jiao [40] identified 13 compounds in the air-dried fruiting body of *F. luteovirens*, 9 of which were identified from this mushroom for the first time. These compounds were identified as D-mannitol (34 mg), 5′-deoxy-5′-methylthioadenosine (23 mg), 5′-deoxy-5′-methylaminoadenosine (18 mg), guanosine (12 mg), adenosine (30 mg), uridine (26 mg), nicotinic acid (26 mg), succinic acid (14 mg), di (2-ethylhexyl) phthalate (15 mg), dibutyl phthalate (23 mg), 3β-linoleoyloxyergosta-7,22-diene (56 mg), 3β, 5α-dihydroxy- (22E, 24R) -ergosta-7,22-dien-6-one (38 mg), and 24-methylcholesta-7,22-diene-3β, 5α, and 6β-triol (21 mg). Pharmacologically, adenosine supports cellular energy metabolism and signal transduction [41,42], while nicotinic acid (vitamin B_3_) supports lipid metabolism and antioxidant defense [43].

Ma Lin [44] studied the chemical constituents in the water extract of the fruiting body of *F. luteovirens*. Seven compounds were isolated and identified as pyroglutamic acid, uridine, 2′-deoxyuridine, uracil, guanosine, inosine, and adenosine. Seven compounds were isolated from the fruiting body of *F. luteovirens* for the first time, and several (e.g., adenosine, guanosine, and uridine) possess immunomodulatory effects and mediate nucleic acid metabolism [45].

Tang Chuchen [46] analyzed the fat-soluble components of the acetone extract of the fruiting body of *F. luteovirens*. A total of 33 compounds were identified, accounting for 98.8% of the total extract content, of which linoleic acid content was as high as 48.2%. Linoleic acid is a key polyunsaturated fatty acid with proven anti-inflammatory and cardiovascular protective activities. The remaining components with more than 2% content were butyl octyl phthalate (12.1%), cis-11-octadecenoic acid (7.1%), mono (2-ethylhexyl) phthalate (7.3%), palmitic acid (4.1%), E-14-hexadecenal (3.3%), E-3-eicosene (3.0%), trans-13-octadecenoic acid (2.6%), and 1-docosadiene (2.1%). Palmitic acid contributes to membrane structure/energy supply [47]; unsaturated fatty acids like cis-11-octadecenoic acid may modulate inflammation [48] (Table 5).

### 3.4. Total Fatty Acids and Phenols

Wang W.E. [49] used supercritical CO_2_ extraction to analyze the products of *F. luteovirens* by GC-MS. The composition and content of 25 fatty acids were separated and identified. The relative content of linoleic acid was 10.6%, and the relative content of monounsaturated fatty acids was 31.5%. Including trans-oleic acid, cis-oleic acid, trans-10-hydroxy-6-methoxy-octadecenoic (10) acid, cis-10-hydroxy-6-methoxy-octadecenoic (10) acid, trans-11-hydroxy-5-methoxy-octadecenoic (11) acid, cis-11-hydroxy-5-methoxy-octadecenoic (11) acid, 7-methoxy-11-hydroxy-eicosenoic (11) acid, and 6-methoxy-12-hydroxy-eicosenoic (12) acid, the relative content of saturated fatty acids accounted for 56.9%. The phenolic acids extracted from the mycelium of *F. luteovirens* by Chen Qihe [50,51,52] and others were identified by HPLC. It was found that the phenolic acids mainly contained ferulic acid, p-coumaric acid, ethyl 4-hydroxybenzoate, 4-hydroxybenzoic acid, and so on. The total phenolic content was 4.21 ± 0.06 mg/g.

### 3.5. Other Active Ingredients

In addition to the aforementioned chemical constituents (polysaccharides, volatile oils, and fatty acids) in the fruiting bodies of *F. luteovirens*, the fruiting bodies also contain a variety of other bioactive substances, which can be classified into the following subcategories:

- Flavonoids: a rich content of flavonoids has been reported in the fruiting bodies [53]; Bai Shijun et al. [16] also confirmed the presence of flavonoids (alongside other constituents).
- Terpenoids: this category includes proto-irane sesquiterpene aryl esters, which have been identified in relevant studies [54,55,56].
- Nitrogen-containing compounds: ergothionein [57]; riboflavins (vitamin B_2_) [58,59]; nucleosides [60]; alkaloids (abundant content) [16].
- Proteins and Enzymes: lectins [61,62,63]; ribonucleases [61,62,63]; fibrinolytic enzymes [64,65].
- Peptides: active peptides are present as important bioactive substances [66,67,68,69].
- Sterols and Steroid-derived compounds: sterols [60]; steroid triterpenes [16].
- Glycosides: this subcategory covers cardiac glycosides, glycosides, and saponins [16].
- Organic acids: a small amount of organic acids has been detected [16].
- Esters and Alkenes: both esters and alkenes are included in the bioactive substances of the fruiting bodies [46].
- Tannins: a small amount of tannins has been reported [16].

In summary (Table 6), *F. luteovirens* contains a variety of functional active substances, which constitute the unique biological activity basis of *F. luteovirens.* It is the existence of these active substances that gives *F. luteovirens* a very high application value. Whether in the research and development of new drugs in the field of biomedicine or in the development of natural health care products, *F. luteovirens* has shown great potential, providing valuable resources for human health and related industrial development.

## 4. Pharmacological Characteristics of *F. luteovirens*

### 4.1. Antioxidant Effect

The results showed that *F. luteovirens* had good scavenging ability on DPPH, ABTS, •OH, and O_2_^−•^free radicals (Table 7). Wang et al. [70] showed that the water extract of *F. luteovirens* (FLPs) had strong antioxidant activity in vitro and good •OH and O_2_^−•^free radical scavenging ability. Xiao et al. [71] found that *F. luteovirens* showed a high DPPH free radical scavenging rate of 65 ± 0.46%. Wu Mengyuan et al. [72] showed that the DPPH free radical scavenging rate and ferrous ion chelating ability of different concentrations of *F. luteovirens* extracts increased in a concentration-dependent manner in the concentration range of 1~5 mg/mL. It has a certain scavenging ability on ABTS free radicals. The results of Qin [52] showed that the IC50 of DPPH free radical scavenging rate was 43.85 μg/mL, the ABTS^+^ scavenging rate was 7.81 mmol/g Trolox, and the FRAP iron ion reduction ability was 1.58 mmol/g FeSO_4_. Zhang et al. [54] determined the DPPH and hydroxyl radical scavenging rates of proto-ilurane sesquiterpene aryl esters in *F. luteovirens*, and the results showed that the DPPH radical scavenging rate was higher. The hydroxyl radical scavenging rate increased with the increase in sample concentration, and the scavenging effect was significantly better than that of ascorbic acid (positive control). Compared with the DPPH free radical scavenging rate of Morchella esculenta [73], the scavenging ability of *F. luteovirens* was not prominent. Wang Huan [35,74] also studied the antioxidant effect of *F. luteovirens* polysaccharide on diabetic rats. The results showed that feeding high, medium, and low dose groups of *F. luteovirens* polysaccharide could alleviate the weight loss of diabetic rats, and the weight had a slight upward trend. It can increase the consumption of exogenous glucose. Studies have shown that edible fungi have long been used as a drug for the treatment of diabetes [75], so *F. luteovirens* will also be able to provide a new area for research and development to control diabetes. Tang et al. [53] used non-targeted and targeted metabolomics to observe the positive correlation between antioxidant activity and flavonoid content in three different regions of *F. luteovirens* in Qinghai and showed that the difference in antioxidant activity of *F. luteovirens* may be mainly attributed to the biosynthesis and metabolism of phenylalanine. Liu et al. [11] obtained two EPS components (ALF1 and ALF2) from the liquid fermentation culture mycelium of *F. luteovirens*. The results showed that the antioxidant activity of ALF1 was better than that of ALF2. ALF1 has good biological activities, such as anti-cancer and antioxidant activities; in addition, ALF1 increased the activities of SOD, GSH-Px, and CAT and reduced the production of MDA, thereby protecting PC12 cells from H_2_O_2_-induced oxidative stress. Funa et al. [25] showed that carboxymethylation and selenization modification could improve the antioxidant and hypoglycemic activities of polysaccharides from *F. luteovirens*. Ma Lin [60] tested the biological activity of the water extract and acetone extract of the fruiting body of *F. luteovirens*. The data showed that the water extract and acetone extract of the fruiting body had significant antioxidant functions, and the acetone extract had a good inhibitory effect on the growth of liver cancer cells. Zhao et al. [76] explored the protective effect and mechanism of FBA on UVA-induced oxidative damage in human skin fibroblasts (HSFs). The results showed that FBA effectively alleviated UVA-induced skin photoaging by enhancing antioxidant components (polysaccharides and peptides), regulating the MAPK/AP-1 pathway, inhibiting MMP activity, and promoting collagen synthesis. These studies have systematically elucidated the antioxidant mechanism of *F. luteovirens.* Although its ability to scavenge DPPH free radicals is not significant, it can contribute to the field of diabetes mellitus. Moreover, the geographical environment has a significant effect on its key metabolites, which can also lay a theoretical foundation for its application.

### 4.2. Immunoregulation

Liu Yan [77,78] used an in vitro gastrointestinal digestion and fermentation system and RAW-blue^TM^ and RAW-264.7 macrophage models to track the changes in immunomodulatory activity of β-glucan and α-glucan in the digestion process of *F. luteovirens*, respectively. It was found that both of them could activate macrophages and increase the proliferation, phagocytosis, and cytokine secretion of macrophages during digestion. The author also compared the effects of different doses (1 mg/kg, 10 mg/kg) of β-glucan and α-glucan (1 mg/kg, 10 mg/kg) on the immune function of immunosuppressive mice induced by cyclophosphamide by gavage and intraperitoneal injection. It was found that low-dose intraperitoneal injection of α-glucan was beneficial to its immune regulation and could cause the body weight of mice to recover quickly and improve the thymus index and immune cell activity of mice and the level of immune factors in serum. Ma [79] systematically revealed the immune regulation mechanism of *F. luteovirens* polysaccharide FLP1 for the first time and provided a theoretical basis for the development of active components of Tibetan medicinal fungi. In addition, through fecal microbiota transplantation (FMT), it was found that [80] *F. luteovirens* polysaccharide affected the intestinal immune function of immunosuppressed mice by adjusting the intestinal flora and could be used as a prebiotic to combat immunosuppression. Zhang et al. [81] found that *F. luteovirens* can improve DSS-induced colitis. The results showed that *F. luteovirens* could effectively reduce the body weight loss and increase the disease activity of mice induced by DSS and improve the colon shortening and colon weight/length ratio induced by DSS. At the same time, it can also reduce the degree of crypt structure damage, alleviate the degree of inflammatory cell infiltration, and reduce the loss of goblet cells. Liu Yang’s [26] studies have shown that the immune regulation function of *F. luteovirens* polysaccharide (FLP) has two-way integrity: by enhancing the function of immune organs, up-regulating pro-inflammatory cytokines and CRP, and activating anti-tumor immune response and by increasing the immune organ index, optimizing the cytokine network (up-regulating beneficial factors and down-regulating excessive inflammatory factors), and regulating the expression of immune genes, the basic immune function is enhanced, and the homeostasis is maintained.

**Figure 3:**
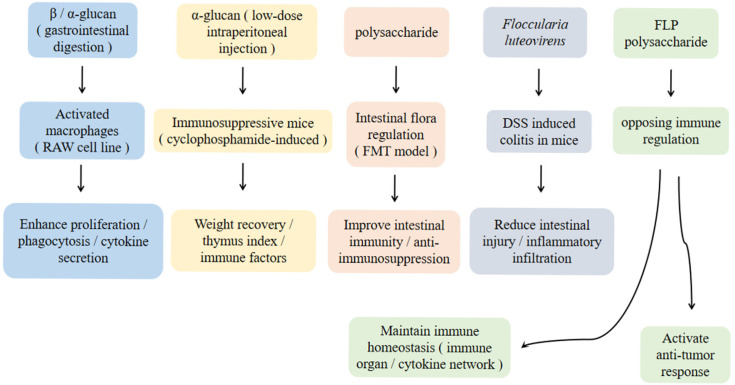
Immune regulation mechanism diagram.

The immune regulatory network of polysaccharides from *F. luteovirens* was analyzed at the molecular, cellular, and even overall levels (Figure 3). It has the characteristics of multi-targets. It can be combined with omics to deeply understand how polysaccharides from *F. luteovirens* achieve immune regulatory functions, so as to provide a theoretical basis for the development of immunomodulators. However, it is necessary to further clarify the relationship between polysaccharide configuration differences and immune effects and establish a clinical transformation research model.

### 4.3. Biocatalysis and Plant Regulation

It also catalyzes the synthesis of some active substances [82,83,84], and its own volatile organic compounds can also regulate plant growth. As a symbiotic fungus, *F. luteovirens* establishes mutualistic relationships with specific host plants, primarily Gastrodia elata (tianma) and Brassica rapa var. chinensis (Chinese cabbage-type rape). Zhang Haifeng [85] showed that F.luteo-virens had a strong ability to produce gastrodin, a key bioactive component in Gastrodia elata, through a biotransformation pathway, where UDP-glucosyltransferase (UGT) catalyzes the glycosylation of p-hydroxybenzyl alcohol using UDP-glucose as the sugar donor. This enzyme exhibits optimal activity at 30 °C and pH 6–7, contributing to a conversion rate as high as 85.2%. A feasible conversion system was established based on these enzymatic characteristics [86,87].

Betulinic acid can also be biocatalytically synthesized by *F. luteovirens*. Liu Jing et al. [88] constructed a biocatalytic system for the synthesis of betulinic acid using laccase as the catalyst, which oxidizes betulin at the C-28 position with the assistance of mediators under optimal conditions of pH 2.0–5.0 and 25–30 °C. The conversion rate can reach as high as 95% by resting cell transformation in an optimized ionic liquid two-phase system. The yield of the optimized system reached 9.32%, which was 74.53% higher than that before optimization.

Feng et al. [89] have shown that gastrodin synthesized by *F. luteovirens* has a good anti-inflammatory effect. Ding [90,91] reported that their volatile organic compounds (VOCs) could regulate rape seedling growth through auxin signaling pathways by inhibiting PIN-FORMED 2 (PIN2) protein abundance, leading to auxin accumulation, which in turn, increased the fresh weight of rape seedling leaves by 34% and 58% and dry weight by 30% and 34%, respectively. Moreover, F. luteo-virens VOCs enhanced carbon/nitrogen metabolism and antioxidant defense systems in rape seedlings, resulting in a 96% increase in soluble sugar content in rape leaves compared with the control. These metabolic changes were accompanied by suppressed secondary metabolism and amino acid catabolism pathways.

Therefore, the study of the biosynthesis systems mediated by *F. luteovirens*, including the UGT-driven gastrodin synthesis and laccase-catalyzed betulinic acid production, as well as the perspective of regulating plant growth through VOC-mediated pathways, can be used as hot spots for future research.

### 4.4. Fresh-Keeping Function

Ma Xuelan’s team [92] and Zhou Lianyu [93] used selenium-enriched fermentation technology to prepare selenium polysaccharide from *F. luteovirens*, which had a significant effect on improving the quality of yogurt: the acid sensory score, pH, acidity, water holding capacity, protein, fat, and mineral element (P, K, Ca, and Fe) content of yogurt were improved. Xu Huimin [94] found that the preservation function of the extracellular polysaccharide of *F. luteovirens* was better: when the concentration of the fungal polysaccharide compound solution was 1.5 g/L and 1.0 g/L, it could better inhibit the color change in the shrimp. It is speculated that this is related to the inhibition of tyrosinase activity and antioxidant activity of the extracellular polysaccharide of *F. luteovirens*. Therefore, the active molecules of *F. luteovirens* also have the function of preservation, which can bring innovative paths in the fields of food preservation and agricultural product storage technology in the future. The specifics are as follows:

(1) Multidimensional expansion in the field of food science and technology: cracking industry pain points

The active molecules of yellow-green L. reevesii are in line with the trend of ‘naturalization, functionalization, and derogation’ and can cover many types of high-demand foods.

a. Upgrading of dairy and plant-based foods

In dairy products, selenium polysaccharide can be extended to cheese, fermented milk beverages, etc., such as low-fat cheese with 0.5~1.0 g/kg selenium polysaccharide, which can replace carrageenan as a natural thickener, improve taste, and supplement selenium (the average daily intake of selenium in Chinese adults is insufficient), matching the needs of ‘nutrition enhancement + clean label’.

b. Fresh agricultural products loss preservation

Fresh meat: extracellular polysaccharide (0.8~1.2 g/L) spray can form a protective film on the surface of fresh meat, extend the shelf life of cold storage from 3~5 days to 7~10 days, reduce fat oxidation rate by more than 30%, and reduce retail loss.

(2) Cooperating with packaging technology: building a new system of ‘active packaging’

This new system breaks through the limitation of traditional packaging using only a physical barrier and realizes the dual function of ‘ barrier + preservation’.

c. Enabled degradable packaging

A coating of 1.0~1.5 g/L extracellular polysaccharides on the inside of biodegradable packaging, such as PLA and PLA, blocks oxygen and water, and polysaccharides slowly release antibacterial and anti-enzymes. For example, the shelf life of pre-cut broccoli is extended from 4~6 days to 10~12 days, and the packaging is degradable within 6 months, balancing preservation and environmental protection.

d. Long-acting controlled release packaging design

The active molecules were made into lipid microcapsules (1~5 μm) and embedded in packaging, which were released slowly with temperature and humidity. For example, after embedding these molecules in vacuum packaging of frozen shrimp, the thawing color retention rate increased by 40%, TVB-N decreased by 25%, and the preservation at−18 °C was more than 6 months, which helped the export of aquatic products.

(3) Commercial value in line with trends

e. Response to consumer demand

Using active molecules matches 78% of consumers’ demand for ‘natural ingredients’ and 65% of consumers’ demand for ‘nutritional fortification’. Active molecules from natural sources can help companies create ‘clean label’ products, such as high-end yogurt. The unit price can be increased by 20~30%.

f. Optimize the supply chain

The shelf life of fresh food is extended by 50−100%, and the energy consumption of the cold chain is reduced by 30%. For example, the return rate of shrimp export is reduced from 8% to less than 2%, which is in line with the goal of ‘double carbon’ and rural revitalization.

### 4.5. Other Resistance Functions

*F. luteovirens* also has anti-tumor [95], anti-inflammatory, anti-aging, and analgesic functions. Wang Huan et al. [96] used a nitroglycerin (NTG)-induced SD rat migraine model as the object and verified the anti-migraine activity of FLW by intragastric administration. The experimental design included five groups (10 male SPF SD rats in each group, weighing 160–200 g): the blank control group (normal saline 1 mL/100g), model group (normal saline 1 mL/100g), positive drug group (rizatriptan benzoate RZB, 1mg/kg, clinical commonly used anti-migraine drugs), FLW low-dose group (150 mg/kg), and FLW high-dose group (300 mg/kg), with continuous administration for 7 days. After 0.5 h of the last administration (except the blank group), NTG (10 mg/kg) was injected subcutaneously to establish the model. After 4 h, the biochemical indexes were detected. The specific verification results were as follows: after high-dose (300 mg/kg) intervention, the levels of serum NO, IL-6, and IL-1β in rats were significantly decreased, and NO was compared with the model group (*p* < 0.001). Chen Qihe and Li Hongji et al. [50,51] reported that the ethanol extract had a significant inhibitory effect on TNF-α secretion in the LPS-induced macrophage inflammation model (*p* < 0.05). Reviewing the anti-inflammatory effects of other edible and medicinal fungi, no studies have clearly shown that other edible fungi have a direct therapeutic effect on migraine. Wang Huan’s study shows that *F. luteovirens* may be the first edible fungus that can be directly used to treat migraine. In the field of anti-migraine active molecules and development of related drugs, *F. luteovirens* are expected to bring new options.

Shao Mingyue [97] used the crude polysaccharide of *F. luteovirens* to treat the mice by gavage. The time of the mouse rotarod test was prolonged to 82.00 ± 6.83 min (model group 33.38 ± 2.50 min), and the swimming time was reduced to 72% (model group); BUN and LA decreased by 36% and 40%, respectively, and SOD activity increased by 85.61 ± 6.35 U/mL (control group 55.0 ± 43.13 U/mL). The results of the applied basic research project ‘Study on the secondary metabolites of *F. luteovirens* and their biological activities’ undertaken by the Northwest Plateau Institute of Biology, the Chinese Academy of Sciences, showed that four components with significant inhibitory activity on the proliferation of liver cancer cells were screened from the secondary metabolites of *F. luteovirens*.

Li Shifeng et al. [95] used *F. luteovirens* from the Qinghai Plateau as materials. After reflux extraction with 80% ethanol, it was extracted with petroleum ether and ethyl acetate in turn to obtain four components: the petroleum ether phase, ethyl acetate phase, residual alcohol-soluble phase, and water-soluble crude polysaccharide phase. The cytotoxicity of human ovarian cancer cells HO-8910 and human liver cancer cells 7721 was detected by the MTT method. The verification results are as follows: *F. luteovirens* had a good cytotoxic effect on human ovarian cancer cell line HO-8910 and human liver cancer cell line 7721. Liu Yang [26] reported that the inhibitory rate of *F. luteovirens* polysaccharide on tumor-bearing mice was 42.48%. These results provide the material basis and technical basis for the application of *F. luteovirens* in the field of anti-tumor research. In the future, more systematic mechanism research can be carried out around its natural anti-tumor active substances that directly kill tumor cells.

**Figure 4:**
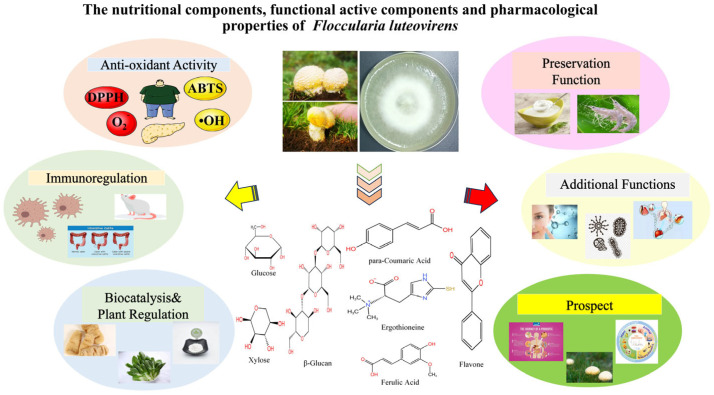
The functions of *F. luteovirens*.

In summary (Table 8), existing studies reveal that *Floccularia luteovirens* has broad application potential in three core fields: edible fungus industrialization, medicinal active substance development, and crop biocontrol and utilization(Figure 4).

- Its active ingredients exert effects through a multi-target regulatory mechanism.
- Its selenium polysaccharides exhibit physical property improvement effects.
- Its polysaccharides can regulate the immune–metabolic regulatory network.
- It can alter the physical and chemical properties of secondary metabolites via biocatalysis.

**Table 8:** Summary of pharmacological characteristics and functional studies of *F. luteovirens*.

Pharmacological Properties	Really Research Content	Key Findings/Findings	Reference
Antioxidation Role	The scavenging ability of DPPH, ABTS, •OH, and O_2_^−^ free radicals.	The scavenging rate of DPPH was 65 ± 0.46%, and the scavenging effect of hydroxyl radical was better than that of ascorbic acid.	[26,36,52,53,54,60,70,71,72,74,75,76]
	Antioxidant and blood glucose regulation effects of polysaccharides on diabetic rats.	Polysaccharides can alleviate the weight loss of diabetic rats and promote glucose consumption.	
	The correlation between antioxidant activity and flavonoid and phenylalanine metabolism in samples from different regions; antioxidant and cytoprotective effects of EPS fractions (ALF1 and ALF2) from liquid fermentation mycelia.	Antioxidant activity was positively correlated with flavonoid content, which was affected by phenylalanine metabolism. ALF1 reduces MDA to protect cells by increasing the activities of SOD, GSH-Px, and CAT.	
	The antioxidant and anti-cancer cell activities of the aqueous extract and acetone extract of the fruiting body; protective mechanism of fermentation broth (FBA) on skin photoaging.	Acetone extract inhibited the growth of liver cancer cells; FBA alleviates photoaging by regulating the MAPK/AP-1 pathway.	
Immunization Regulating	The activation of macrophages by β-glucan and α-glucan during digestion; effects of different doses of dextran on immune function (body weight, thymus index, immune factors) in immunosuppressed mice.	Glucan activates macrophages, enhances proliferation, phagocytosis, and cytokine secretion; low-dose α-glucan intraperitoneal injection has the best effect and promotes weight recovery.	[27,77,78,79,80,81]
	Polysaccharides affect intestinal immunity in immunosuppressed mice by adjusting intestinal flora; improvement of DSS-induced colitis (body weight, colon structure, and inflammatory cell infiltration).	As a prebiotic, polysaccharides can regulate intestinal flora and improve colitis.	
	The bidirectional immune regulation of polysaccharides (enhancing anti-tumor immunity and maintaining basic immune homeostasis).	It has a multi-target immune regulation network, and the relationship between polysaccharide configuration and immune effect needs to be further studied.	
Biocatalysis and Plant Regulation	Gastrodin synthesis ability and transformation system; biocatalytic synthesis of betulinic acid; anti-inflammatory effect of synthetic gastrodin.	The conversion rate of gastrodin was up to 85.2%, and the conversion rate of betulinic acid was 95%.	[82,83,84,85,86,87,88,89,90,91]
	Effects of volatile organic compounds (VOCs) on the growth of rapeseed.	VOCs significantly increased the fresh weight, dry weight, and soluble sugar content of rape seedlings.	
Preservation Functions	Effect of selenium-enriched fermentation preparation of selenium polysaccharide on the quality of yoghurt.	Improve a number of yogurt quality indicators (pH, protein, mineral elements, etc.).	[92,93,94]
	Preservation effect and mechanism of extracellular polysaccharide compound solution on shrimp.	The 1.5 g/L and 1.0 g/L polysaccharide solutions could inhibit the color change in shrimp, which was related to the inhibition of tyrosinase and antioxidant activity.	
Other Resistance Functions	Anti-tumor, anti-inflammatory, anti-migraine, anti-aging, analgesic, and other functions.	The water extract significantly reduced the levels of inflammatory factors such as NO and IL-6 in the serum of migraine rats; ethanol extract inhibited TNF-α secretion of macrophages.	[50,95,96,97]
		Polysaccharides had cytotoxic effects on liver cancer and ovarian cancer cells, and the tumor inhibition rate of tumor-bearing mice was 42.48%. The crude polysaccharide prolonged the exercise endurance of mice and increased SOD activity.	

## 5. Outlook

### 5.1. Short-Term Goals

#### 5.1.1. Multi-Omics Analysis of Bioactivity Mechanisms

With the progress of omics technology, the internal mechanism of the active components of *F. luteovirens* can be studied by omics, and the influence of genetic information on the synthesis pathway of bioactive substances such as polysaccharides can be studied by genomics. Through transcriptomics, we can identify genes that play a key regulatory role in different growth environments; through proteomics and metabolomics, the changes in proteins and metabolites can be directly elucidated, and the precise relationship between components and efficacy can be established.

#### 5.1.2. Preliminary Optimization of Artificial Domestication

At the molecular level, the associations between gene expression and external factors (e.g., nutrients, micro-ecological environment) affecting mycelial growth should be refined to identify core regulatory factors. Specific transcription factors should be studied to modulate mycelial growth rate and stress resistance for cultivating high-quality mycelia.

#### 5.1.3. Targeted Investigation of Medicinal Mechanisms

It is known that the *F. luteovirens* has a variety of medicinal values, but the specific mechanism of action has not been fully understood. Taking immune regulation as an example, the interaction between polysaccharides and immune cells in the intestine is complex and can be promoted by activating and regulating different signaling pathways. The research on the synergistic mechanism between different medicinal active ingredients in *F. luteovirens* should also be carried out. On the basis of understanding the molecular mechanism of polysaccharides activating immune cells alone, combined with the common antioxidant, anti-inflammatory, and immunomodulatory effects of polysaccharides, flavonoids, and polyphenols, the synergistic mechanism should be explored to prepare for the subsequent development of effective compound drugs.

#### 5.1.4. Foundational Development of Functional Products

We recommend using advanced separation and purification techniques (e.g., high-speed counter-current chromatography and preparative liquid chromatography) to obtain high-purity active ingredients. We recommend investigating the structure–activity relationships of these purified active ingredients.

### 5.2. Long-Term Goals

#### 5.2.1. Breakthroughs in Artificial Domestication and Large-Scale Cultivation

Drawing on comparative studies of domestication in model edible fungi (e.g., Lentinula edodes and Morchella esculenta), multi-omics-driven comparative analyses can be employed to decipher the molecular basis of *F. luteovirens* domestication and enrich the mechanistic details:

a. Comparative genomics with domesticated relatives: Sequence *F. luteovirens* genome and compare it with closely related domesticated species (e.g., *Armillaria mellea*) to identify domestication-associated genomic signatures (e.g., expanded gene clusters for nutrient assimilation, stress tolerance, or fruiting body development). Leverage insights from Morchella domestication (amplified CAZyme gene families for plant substrate breakdown) to pinpoint *F. luteovirens* CAZyme genes critical for utilizing agricultural byproducts (e.g., corn stover, wheat straw).
b. Transcriptomic dissection of mycelial growth and primordium formation: Conduct time-course transcriptomics during mycelial proliferation and primordium initiation. Compare gene expression between wild-type *F. luteovirens* (low domestication potential) and lab-adapted strains (improved growth/fruiting). We recommend focusing on the following:
  ◆ Transcription factors (TFs) regulating hyphal branching (e.g., Flo8 homologs) and upstream signaling (e.g., MAPK pathways for environmental sensing).
  ◆ Metabolic genes for trehalose biosynthesis (mycelial stress protection) or polyketide synthesis (primordium differentiation, as in Coprinopsis cinerea).
c. Functional validation of key regulatory nodes: use genetic tools (e.g., CRISPR-Cas9 and RNA interference) to verify candidate genes/pathways:
  ◆ Overexpress trehalose-6-phosphate synthase (from transcriptomics) to enhance mycelial tolerance to suboptimal temperatures.
  ◆ Knock down repressor TFs of primordium formation to accelerate fruiting body development.

#### 5.2.2. Synergistic Research on Medicinal Mechanisms

We recommend investigating how multiple active ingredients (polysaccharides, flavonoids, and polyphenols) synergistically exert antioxidant, anti-inflammatory, and immunomodulatory effects. The groundwork for developing effective compound drugs needs to be laid.

#### 5.2.3. Advanced Development of Functional Foods and Pharmaceuticals

We recommend conducting structural modification of active ingredients via chemical synthesis or biotransformation to enhance biological activity and pharmacokinetic properties (e.g., modifying polysaccharides to improve antioxidant capacity and bioavailability). High-value-added functional foods and drugs based on optimized active ingredients should be developed.

#### 5.2.4. Interdisciplinary Translation of Research

We recommend strengthening cross-disciplinary collaboration with fields such as ecology, medicine, and food science to translate *F. luteovirens* basic research into practical applications, maximizing its ecological, economic, and social value.

**Figure 5:**
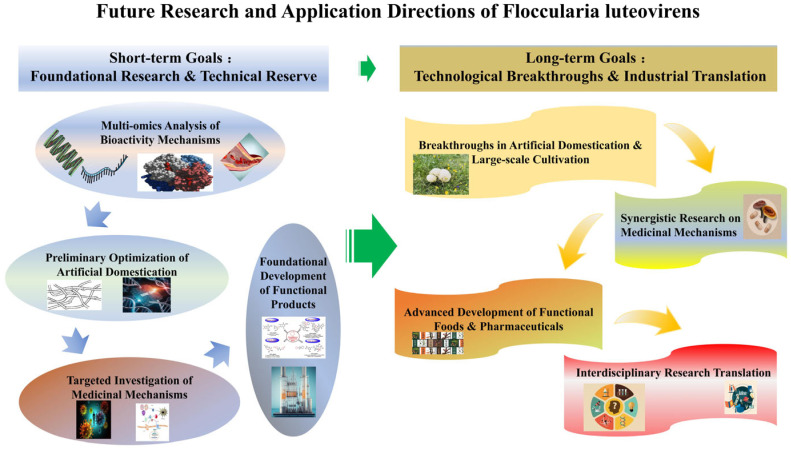
Future research and application directions of *Floccularia luteovirens*.

In summary (Figure 5), *F. luteovirens* has broad prospects in the application of multi-omics, ecological functions, and interdisciplinary fields; however, there are still some shortcomings and contents to be explored in the research of artificial cultivation, medicinal mechanism, and component development, which need to be explored by subsequent researchers. The future research can focus on the above problems, strengthen the interdisciplinary research with other disciplines, promote *F. luteovirens* with respect to basic research to practical use, and give full play to the ecological, economic, and social value of *F. luteovirens*.

## Figures and Tables

**Table 1:** Specific types and contents of 19 amino acids.

Amino Acid	Content (mg/g)	Amino Acid	Content (mg/g)	Amino Acid	Content (mg/g)	Amino Acid	Content (mg/g)
methionine	0.16~0.35	leucine	0.41~0.81	asparagine	5.72~9.29	alanine	2.23~11.94
tryptophan	9.65~21.62	phenylalanine	0.03~1.78	serine	2.05~3.88	proline	3.91~9.49
lysine	0.03~1.33	threonine	3.43~5.86	glycine	2.23~3.56	tyrosine	0.57~1.06
valine	0.48~1.29	aspartic acid	1.51~2.24	histidine	0.46~2.55	cysteine	0.15~0.20
isoleucine	0.01~0.05	glutamic acid	6.42~7.90	arginine	5.33~10.33		

**Table 2:** Mineral types and contents (g/kg) of *F. luteovirens*.

Collection Sites	Ca	Cu	Fe	K	Mg	Mn	Na	P	S	Zn
Qilian A	0.66	0.04	0.24	32.43	1.66	0.02	0.22	10.93	5.65	0.09
Qinghai Lake	0.50	0.049	0.32	30.95	1.66	0.02	0.19	10.75	5.10	0.09
Yushu	0.42	0.044	0.15	31.39	1.68	0.01	0.26	10.97	4.74	0.82
Geermu City	0.62	0.05	0.26	32.21	1.85	0.02	0.20	11.89	5.13	0.09
Qilian B	0.40	0.05	0.18	28.61	1.59	0.013	0.21	10.40	4.62	0.09

**Table 4:** Comparison of extraction, purification, and fermentation methods for *F. luteovirens* polysaccharides.

Researcher	Target Material	Method Type	Key Process Parameters	Polysaccharide Outcome (Yield/Purity)	Reference
Du et al.	Fruiting bodies	Ultrasonic-assisted extraction	Optimized ultrasonic conditions	Extraction yield: 7.93%	[29]
Shi Qang qiang	Mycelia	Purification (DEAE-52 cellulose column)	DEAE-52 cellulose chromatography for separation/purification	Purity: >99%	[30]
Liu Yang	Polysaccharide extract	Detection (HPLC)	HPLC for β-glucan and mannose oligosaccharides quantification	β-glucan: 20.1%; mannose oligosaccharides: 5.7% (content in extract)	[26]
Dang Jun	Mycelia	Water extraction	Water extraction; phenol-sulfuric acid method for content determination	Content in water extract: 31.21%	[31]
Tao Yanduo	Cultured mycelia	Fermentation + extraction	Mycelium culture; polysaccharide extract production process	Content in extract: >53%; mycelium polysaccharide content: 31%; yield: 2.3%	[32]
Wang Hong	Extracellular polysaccharides (EPSs)	Fermentation (medium optimization)	Optimal medium: potato 20 g, glucose 4 g, yeast extract 0.20 g, KH_2_PO_4_ 0.15 g, MgSO_4_ 0.05 g/100 mL, VB_1_ 12 μg/mL, pH 6.0	EPS production (medium optimization, no direct yield reported)	[33]
Xiao Qianqing	Mycelia	Extraction + fermentation (process optimization)	Optimized extraction and fermentation conditions	Extraction yield: 11.86% (highest reported)	[34]
Liu et al.	Fruiting bodies	Hot water extraction + purification	Hot water extraction (80 °C); Sevag method (deproteinization); H_2_O_2_ (decolorization); 95% ethanol (precipitation)	Crude polysaccharide yield: 1.72% (lowest reported)	[27]
Wang Huan et al.	Crude polysaccharide	Composition analysis	Extraction/separation of crude polysaccharide; phenol-sulfuric acid method	Total sugar content: 65.0%; reducing sugar content: 7.2%	[35]

**Table 5:** Bioactivities of major bioactive substances among other compounds in *F. luteovirens* fruiting bodies.

Compound Name	Source	Bioactivities	Reference
Adenosine	Jiao [40]; Ma [44]	Regulates cellular energy metabolism and signal transduction; exerts immunomodulatory effects; mediates nucleic acid metabolism	[41,42,45]
Guanosine	Jiao [40]; Ma [44]	Exhibits immunomodulatory effects; mediates nucleic acid metabolism	[45]
Uridine	Jiao [40]; Ma [44]	Exhibits immunomodulatory effects; mediates nucleic acid metabolism	[45]
Nicotinic acid (vitamin B_3_)	Jiao [40]	Supports lipid metabolism; enhances antioxidant defense	[43]
Linoleic acid	Tang [46]	Possesses anti-inflammatory activity; confers cardiovascular protection	(text)
Palmitic acid		Contributes to membrane structure; supplies energy	[47]
cis-11-octadecenoic acid		May modulate inflammation	[48]

**Table 6:** List of functional active substances and their characteristics of *F. luteovirens*.

Active Substance Category	Specific Composition and Content	Extraction Methods /Research Findings	Pharmacological Activity/Function	Reference
Polysacc- haride	Monosaccharides: glucose, mannose, fucose, rhamnose, galactose, xylose, arabinose.	Ultrasonic extraction, water extraction, organic solvent extraction, column separation (DEAE-52 cellulose chromatography column, purity > 99%).	Anti-inflammatory; analgesia; antioxidation; anti-tumor; anti-aging; immune regulation	[25,27,28]
	Polysaccharides: β-glucan (20.1%), mannan oligosaccharides (5.7%).			[26]
	Total sugar content: 65.0% (crude polysaccharide), reducing sugar 7.2%.	Purification steps: Sevag method to remove protein, H_2_O_2_ decolorization, ethanol precipitation.		[15,35]
	Extraction rate: ultrasound-assisted extraction of the highest 11.86%, water extract polysaccharide content of 31.21%~53%.			[31,32]
VOCs	Main components: alkanes (such as 2-methyl eicosane 12.23%, n-heptadecane 6.24%), esters (linoleic acid methyl ester 10.94%), terpenes (zingiberene 9.10%, bergapten 4.62%), ketones (2-nonanone 3.41%).	Headspace solid-phase microextraction–gas chromatography–mass spectrometry (SPME-GC-MS) identification.	Strong aroma, potential antioxidant, antibacterial, and other activities (speculated)	[36,37]
	Characteristic components: C15 terpenes, C14-C18 alkane isomers, C8-C13 ketene/aldehyde/alcohol.	The source of fruiting body fragrance: terpenes, alkanes, ketene/aldehyde/alcohol compounds.		[37]
Fruiting body compounds	Nucleosides: adenosine (5.30 mg), guanosine (4.12 mg), uridine (6.26 mg).	Nine compounds (such as 5′-deoxy-5-methylthioadenosine) were identified from this mushroom for the first time. Seven new compounds, such as pyroglutamic acid and uracil, were isolated from the water extract.	Regulation of metabolism, antioxidation, and potential medicinal value (further research is needed)	[40]
	Alcohols/ketones: 3β-linoleoyloxyergosta-7,22-diene(56 mg), 3β,5α-dihydroxy; acids: succinic acid (8.14 mg), nicotinic acid (7.26 mg).			
	Esters: phthalates (9.15~10.23 mg).			
fatty acid	Unsaturated fatty acids: linoleic acid (48.2%~10.6%), cis-oleic acid, trans-oleic acid.	Supercritical CO_2_ extraction +GC-MS analysis; linoleic acid is the main component of fat-soluble components.	Regulate blood lipids, anti-inflammatory, antioxidant (unsaturated fatty acids dominate)	[49]
	Saturated fatty acids: 56.9%.			
	Special structure: containing methoxy hydroxy olefinic acid (such as 10-hydroxy-6-methoxyoctadecenoic acid).			
phenols	Main phenolic acids: ferulic acid, p-coumaric acid, 4-hydroxybenzoic acid, and its esters.	Identification by HPLC, extraction from mycelium.	Antioxidant, anti-inflammatory, antibacterial	[50,51,52]
	Total phenolic content: 4.21 ± 0.06 mg/g			
Other Ingredients	Flavonoids, protoluane sesquiterpene aryl ester, ergothioneine, lectin, active peptide, riboflavin, fibrinolytic enzyme, sterols, nucleosides, esters, alkenes.	Alkaloids/flavonoids were identified by the chemical coloration method. Ergothioneine: a substance with high antioxidant activity.	Antioxidation, immune regulation, antithrombosis (plasmin), regulation of metabolism (nucleosides), etc.	[46,53,54,55,56,57,58,59,60,61,62,63,64,65,66,67,68,69]
	Containing alkaloids, flavonoids, cardiac glycosides, steroid triterpenoids, saponins, etc.			[16]

**Table 7:** Antioxidant activities of *F. luteovirens* vs. control mushrooms.

Sample Type	Assay	Activity Metric	Value	Control/Comparison	Reference
*F. luteovirens* water extract (FLPs)	•OH scavenging	Relative activity	Potent	Different extraction processes for polysaccharide	[70]
	O_2_^−•^ scavenging	Relative activity	Potent		
*F. luteovirens* (whole mushroom)	DPPH scavenging	Rate	65 ± 0.46%	different processing methods	[71]
*F. luteovirens* extract	DPPH scavenging	IC_50_	43.85 μg/mL	Different extraction processes of phenols	[52]
	ABTS^+^ scavenging	Trolox equivalent	7.81 mmol Trolox/g		
	FRAP	FeSO_4_ equivalent	1.58 mmol FeSO_4_/g		
*F. luteovirens* proto-ilurane sesquiterpene aryl esters	•OH scavenging	Relative to ascorbic acid	More potent than ascorbic acid	Ascorbic acid (positive control)	[54]
*Morchella esculenta*	DPPH scavenging	Rate	Higher than *F. luteovirens*	*F. luteovirens*	[54,72]

## Data Availability

No data was used for the research described in the article.
